# Association between the non-high-density lipoprotein cholesterol to high-density lipoprotein cholesterol ratio (NHHR) and cardiovascular outcomes in patients undergoing percutaneous coronary intervention: a retrospective study

**DOI:** 10.1186/s12944-024-02309-4

**Published:** 2024-10-01

**Authors:** Jiuling Liu, Melysze Deanne Oorloff, Adithya Nadella, Ping Guo, Min Ye, Xiaoqing Wang, Hang Zhao

**Affiliations:** 1 Department of Neurology, Nanjing BenQ Medical Center, the Affiliated BenQ Hospital of Nanjing Medical University, Nanjing, Jiangsu Province China; 2 Nanjing Medical University, Nanjing, Jiangsu Province China; 3 Department of Cardiology, Nanjing BenQ Medical Center, the Affiliated BenQ Hospital of Nanjing Medical University, Nanjing, Jiangsu Province China; 4 Shanghai Medical College, Fudan University, Shanghai, China; 5 Department of Anesthesiology, The Yancheng School of Clinical Medicine of Nanjing Medical University (Yancheng Third People’s Hospital), Yancheng, Jiangsu Province China

**Keywords:** Coronary artery disease, NHHR, MACCEs

## Abstract

**Background:**

Dyslipidemia is prominently associated with adverse outcomes in patients with coronary artery disease (CAD). The non-high-density lipoprotein cholesterol to high-density lipoprotein cholesterol ratio (NHHR) is a novel comprehensive lipid index. However, limited evidence exists on the relationship of the NHHR with the risk of adverse outcomes in patients with CAD. This study aimed to explore the associations between the NHHR and adverse outcomes and identify the optimal NHHR ranges linked to the lowest adverse outcome risk in patients with CAD undergoing percutaneous coronary intervention (PCI).

**Methods:**

Among 2253 patients with CAD undergoing PCI, 2251 with available total cholesterol and HDL-C levels were analyzed. Furthermore, all patients were classified into quintiles based on the NHHR. The primary outcome was the incidence of MACCEs, comprising cardiac mortality, acute myocardial infarction, stroke, and repeat revascularization. Multivariable logistic regression analysis was used to assess the relationship between the NHHR and MACCEs. Moreover, restricted cubic spline (RCS) analysis was performed to quantify nonlinearity. Lastly, the consistency between these associations was confirmed by conducting subgroup and interaction analyses.

**Results:**

A total of 270 patients experienced MACCEs over a median follow-up of 29.8 months (interquartile range, 25.6–34 months). After adjustment for confounding variables, the adjusted ORs (95% CIs) of the patients in quintiles 2, 3, 4, and 5 were 0.79 (0.52–1.20), 0.64 (0.42–0.99), 1.00 (0.67–1.48), and 1.17 (0.74–1.64), respectively (reference group: quintile 1). Additionally, RCS analysis demonstrated a U-shaped relationship between the NHHR and MACCEs, with an inflection point at an NHHR of 3.119 using a two-piecewise regression model. This relationship was consistent across the various subgroups, while significant interactions were not observed in these associations.The ORs and 95% CIs to the left and right of the inflection point were 0.734 (0.551–0.978) and 1.231 (1.038–1.460), respectively.

**Conclusions:**

This study reveals a U-shaped association between baseline NHHR and MACCE incidence in patients with CAD undergoing PCI.

**Supplementary Information:**

The online version contains supplementary material available at 10.1186/s12944-024-02309-4.

## Introduction

Coronary artery disease (CAD) is a prevalent condition that poses a serious threat to human health, accounting for 9 million deaths annually worldwide [1]. Although the application of reperfusion strategies and enhancement of regional coordinated treatment systems have notably reduced acute phase mortality in patients with CAD [2–4], the incidence of major adverse cardiovascular and cerebrovascular events (MACCEs) following percutaneous coronary intervention (PCI) continues to increase [5, 6]. Dyslipidemia is a common condition among patients with confirmed CAD and is associated with adverse outcomes. Therefore, identifying residual risk factors in patients with CAD undergoing PCI is critical for lowering MACCE risk.

Conventionally, low-density lipoprotein cholesterol (LDL-C) has been the primary focus in dyslipidemia management among patients with CAD. However, aggressive LDL-C-lowering treatments are unable to mitigate the heightened risk of residual cardiovascular events in this patient population [7]. The non-high-density lipoprotein cholesterol to high-density lipoprotein cholesterol ratio (NHHR) is a novel comprehensive lipid index of atherogenic lipids, which integrates all atherogenic and anti-atherogenic lipid measurements [8]. The NHHR can be easily obtained from normal lipid profiles in clinical practice at no additional cost [9]. Previous studies have revealed that NHHR is a major risk factor for insulin resistance, nonalcoholic fatty liver disease [10], carotid atherosclerosis [11–13], diabetes [14, 15], hyperuricemia [16], and CAD [17, 18]. Furthermore, a study by Jiayin You et al. on the association between NHHR and CAD progression found a relationship between baseline NHHR and MACCEs [18]. However, the researchers only performed a subgroup analysis using MACCEs as the stratification factor, without accounting for potential confounding factors or assessing dose–response relationships. This limitation does not provide a comprehensive perspective on the precise relationship between the NHHR and MACCE risk. Consequently, the conclusions drawn by Jiayin You et al. were restricted, underlining the necessity for our current study [18]. Thus, this study aims to investigate the association between baseline NHHR and MACCE risk in patients with CAD undergoing PCI by performing a secondary data analysis utilizing existing data from a published source [19].

## Methods

### Study design and patients

**Fig. 1:**
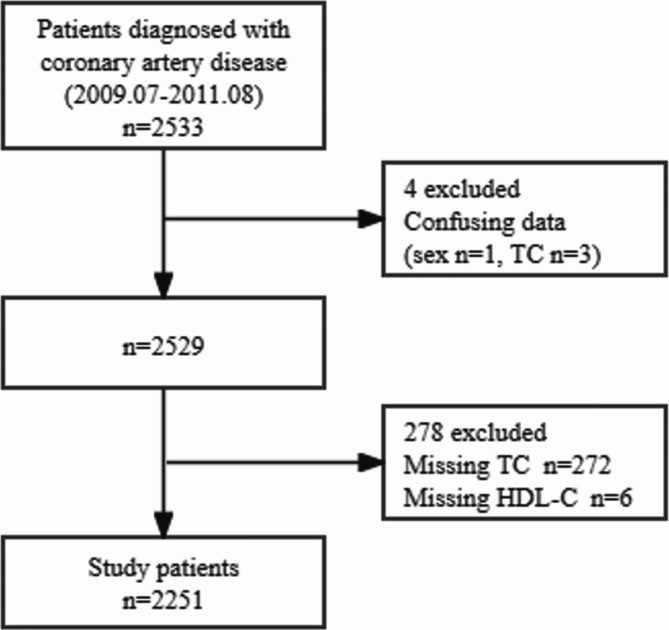
Flowchart of participant selection. Note: TC, total cholesterol; HDL-C, high-density lipoprotein cholesterol

The data for this study were obtained from the “DATADRYAD” database (www.datadryad.org), which allows users to freely download raw data. The Terms of Service of Dryad were followed, and the relevant Dryad data packages were cited in this study [19]. This study was conducted between July 2009 and August 2011 at the First Affiliated Hospital of Zhengzhou University, a high-volume PCI center in China. The study included 2533 patients who underwent PCI at the center using established techniques. Before the coronary intervention, all patients received loading doses of 300 mg of aspirin and 300 mg of clopidogrel, except those already on antiplatelet medications. After PCI, the patients were maintained on standard dual antiplatelet therapy, encompassing a daily dose of 100 mg of aspirin and 75 mg of clopidogrel for at least 1 year. The patients were followed up for a median of 29.8 months (interquartile range: 25.6–34 months). After excluding unclear or missing total cholesterol (TC) and HDL-C data, the data from 2251 patients were finally included **(**Fig. 1**).**

### Ethics approval and consent to participate

The Ethics Committee of the First Affiliated Hospital of Zhengzhou University approved the research protocol. Considering the retrospective nature of this study, the ethics committee waived the requirement for informed consent. This study was conducted in accordance with the guidelines of the Helsinki Declaration. Further details of the study design can be obtained from a previous investigation [19]. Given that the public policy statement of the dataset utilized in this study has already been approved by the ethics committee, the present study did not require any additional ethical declaration.

### Data collection and outcome definition

Information obtained on admission comprised age, sex, smoking status, left ventricular ejection fraction, clinical presentation (ST-segment myocardial infarction [STEMI], non-ST elevation acute coronary syndrome [NSTE-ACS], or stable angina [SA]), and comorbidities (heart failure, atrial fibrillation, prior myocardial infarction [MI], prior stroke, hypertension, and diabetes). The collected angiographic data included various aspects, such as the employed surgical technique, the precise location of target lesions (including the left main coronary artery, left circumflex artery, left anterior descending coronary artery, and right coronary artery), and the number of affected vessels. The lesion characteristics that were recorded entailed whether they were occluded, chronic total occlusions (CTO), located at the ostium, or bifurcation lesions. Furthermore, information on the number of vessels that required treatment and the number, length, and diameter of utilized stents were documented. Data on various drugs such as aspirin, clopidogrel, β-blockers, and statins were also collected. Patient follow-ups were conducted via outpatient visits, readmissions, or telephone communication. Hypertension was defined as the use of antihypertensive medications or a self-reported history of high blood pressure. Diabetes was considered as the use of antidiabetic medications or self-reported diabetes. Individuals who reported smoking within the last decade were classified as smokers.

Laboratory data were obtained from the patients’ medical records. The collected data consisted of a series of standard laboratory tests that measured creatinine, glucose, TC, triglyceride (TG), HDL-C, and LDL-C values from fasting blood samples. The non-HDL-C level was estimated as the difference between serum TC and serum HDL-C concentrations. Finally, the NHHR was determined by dividing the level of non-HDL-C by that of HDL-C.

### Outcome measures

The primary study outcome was the incidence of MACCEs during follow-up. MACCE incidence was defined as the occurrence of cardiac mortality, acute myocardial infarction (AMI), stroke, and repeat revascularization. All patients were followed up from baseline until cardiac death, AMI, stroke, or repeat revascularization or to the censoring date (August 2011), depending on whichever occurred first. Clinical follow-up was conducted via patient visits, telephone interviews, and a retrospective examination of medical records. All data entry was performed by independent researchers, while a separate committee adjudicated clinical events.

### Statistical analysis

Patients were categorized into quintiles based on the NHHR: quintile 1 (*n* = 445, NHHR < 2.15), quintile 2 (*n* = 445, 2.15 ≤ NHHR ≤ 2.77), quintile 3 (*n* = 357, 2.78 ≤ NHHR ≤ 3.39), quintile 4 (*n* = 353, 3.40 ≤ NHHR ≤ 4.22), and quintile 5 (*n* = 392, NHHR > 4.22), as described in prior studies [20, 21]. The chi-square test is applied to test for differences between groups and represented as number (n) and percentage (%). Normally distributed continuous variables were presented as mean ± standard deviation (SD), while non-normally distributed continuous variables were expressed as median (interquartile range). One-way analysis of variance (ANOVA) tests for differences between normally distributed variables and the Kruskal-Wallis test tests for differences between medians.

The association between the NHHR and MACCEs was investigated by applying univariable and multivariable logistic regression models. In all these analyses, quintile 1 (< 2.15) of the NHHR was utilized as the reference group in the separate models. In the initial crude model, no covariables were adjusted. Subsequently, adjustments for age and sex were made in model I. In model II, the adjustments included those for age, sex, smoking status, medical history of diseases (MI, heart failure, atrial fibrillation, stroke, hypertension, and diabetes), use of aspirin, statins, and β-blockers, 3-vessel disease, total stent length, and stent diameter. All these confounders were selected based on their association with the outcomes of interest, clinical significance, identification in the literature, or changes in effect estimates of more than 10% [22, 23].

Next, we conducted a restricted cubic spline (RCS) analysis to illustrate the dose–response relationship between the NHHR and MACCEs after adjusting for the confounding factors as in the logistic regression model II. In cases exhibiting a non-linear association, a two-piecewise logistic regression model was applied to examine the threshold saturation effect of the NHHR on MACCE risk, with adjustments similar to that for the model II variables [24]. The recursive method automatically identified the inflection point on the smoothing curve that produced the maximum model likelihood. The log-likelihood ratio test was further employed to determine the optimal model for assessing the association between the NHHR and MACCEs, as outlined in a preceding analysis [23, 24]. The bootstrap resampling technique was then utilized to compute the 95% confidence interval (CI) for the inflection point, as described in a prior study [25].

Additionally, stratified analyses were conducted to evaluate the heterogeneities in the associations between baseline NHHR and MACCEs across the subgroups of MACCE risk, sex, age (< 65 years vs. ≥65 years), clinical presentation (STEMI vs. SA vs. NSTE-ACS), smoking status (no vs. yes), CTO (no vs. yes), and medical history of diabetes, hypertension, or heart failure. Data analyses were performed using the statistical software packages R and EmpowerStats. The results were presented as odds ratios (ORs) with corresponding 95% CIs. Statistical significance was set at a *p*-value of < 0.05.

## Results

### Baseline characteristics of the study patients

Among the total 2251 patients included in this study, the mean age was 60.0 ± 11.1 years and 718 (31.9%) were men. The baseline characteristics of the study patients categorized according to the NHHR quintiles are presented in Table 1. Patients in the highest NHHR quintile (quintile 5) were more likely to be smokers and have higher TC, TG, and LDL-C levels, heightened percentages of STEMI, and higher proportions of history of diabetes. Patients in the lowest NHHR quintile (quintile 1) were more prone to be older, be of male sex, have NSTE-ACS, exhibit higher HDL-C levels, and have greater percentages of bifurcation lesions (all *P* < 0.05).

**Table 1:** Baseline characteristics of patients included according to NHHR Quintiles

Characteristics	All	Quintiles of NHHR					*P*-value
		Q1 (< 2.15)	Q2 (2.15–2.77)	Q3 (2.78–3.39)	Q4 (3.40–4.22)	Q5 (> 4.22)	
**N**	2251	445	445	457	451	453	
**Age(years)**	60.0 ± 11.1	61.9 ± 11.3	61.1 ± 10.9	60.7 ± 10.7	59.3 ± 10.4	57.1 ± 11.4	< 0.001
**Men**,** n(%)**	718 (31.9)	159 (35.7)	148 (33.3)	162 (35.4)	128 (28.4)	121 (26.7)	0.007
**Smoking**,** n(%)**	739 (32.8)	120 (27)	140 (31.5)	145 (31.7)	162 (35.9)	172 (38)	0.005
**Clinical presentation** 0.098							
STEMI, (%)	567 (25.2)	87 (19.6)	104 (23.4)	126 (27.6)	122 (27.1)	128 (28.3)	
SA, (%)	322 (14.3)	65 (14.6)	68 (15.3)	61 (13.3)	66 (14.6)	62 (13.7)	
NSTE-ACS, (%)	1362 (60.5)	293 (65.8)	273 (61.3)	270 (59.1)	263 (58.3)	263 (58.1)	
**LVEF**,** Mean ± SD**	60.9 ± 7.4	61.5 ± 6.7	61.1 ± 7.6	60.3 ± 7.6	60.9 ± 7.6	60.9 ± 7.6	0.348
**Medical history**,** n(%)**							
Heart failure	269 (12.0)	56 (12.6)	56 (12.6)	58 (12.8)	52 (11.6)	47 (10.4)	0.779
Atrial fibrillation	45 ( 2.0)	12 (2.7)	11 (2.5)	8 (1.8)	7 (1.6)	7 (1.5)	0.610
Prior MI	222 ( 9.9)	46 (10.3)	42 (9.4)	48 (10.5)	42 (9.3)	44 (9.7)	0.965
Prior stroke	118 ( 5.2)	31 (7)	21 (4.7)	29 (6.3)	20 (4.4)	17 (3.8)	0.156
Hypertension	1132 (50.3)	202 (45.4)	229 (51.5)	226 (49.6)	238 (52.8)	237 (52.3)	0.167
Diabetes	488 (21.7)	82 (18.4)	81 (18.2)	110 (24.1)	101 (22.4)	114 (25.2)	0.028
**Laboratory datas**							
Creatinine, umol/L, (IQR)	69.0 (58.0, 81.0)	68.0 (56.0, 82.0)	71.0 (59.0, 83.0)	69.0 (58.0, 80.0)	69.0 (58.0, 80.0)	70.0 (58.0, 80.0)	0.303
Glucose, mmol/L, (IQR)	6.0 (5.3, 6.7)	5.6 (4.7,6.3)	5.9 (4.9,6.6)	5.8 (4.7,6.5)	6.3 (5.0,6.9)	6.2 (5.0,7.6)	0.006
TC, mmol/L	4.3 ± 1.1	3.5 ± 0.8	4.0 ± 0.9	4.2 ± 1.0	4.5 ± 0.9	5.1 ± 1.1	< 0.001
TG, mmol/L, (IQR)	1.6 (1.1, 2.2)	1.1 (0.8, 1.4)	1.4 (1.1, 1.8)	1.5 (1.2,2.1)	1.9 (1.4,2.6)	2.3 (1.6,3.3)	< 0.001
HDL-C, mmol/L	1.1 ± 0.3	1.4 ± 0.3	1.2 ± 0.3	1.0 ± 0.2	0.9 ± 0.2	0.8 ± 0.2	< 0.001
LDL-C, mmol/L	2.7 ± 0.9	2.0 ± 0.7	2.4 ± 0.8	2.7 ± 0.8	2.9 ± 0.8	3.3 ± 1.0	< 0.001
**Treatment**,** n(%)**							
Aspirin	2221 (98.8)	442 (99.3)	440 (98.9)	451 (98.9)	440 (97.8)	448 (98.9)	0.299
Clopidogrel	2158 (95.9)	430 (96.6)	419 (94.2)	441 (96.5)	436 (96.9)	432 (95.4)	0.337
β-blocker	1581 (70.2)	322 (72.4)	310 (69.7)	308 (67.4)	318 (70.5)	323 (71.3)	0.549
Statin	2111 (93.8)	417 (93.7)	415 (93.3)	435 (95.2)	427 (94.7)	417 (92.1)	0.319
**Number of diseased vessels**,** (%)**							
1-vessel disease	878 (39.0)	188 (42.2)	187 (42)	180 (39.4)	166 (36.8)	157 (34.7)	0.084
2-vessel disease	835 (37.1)	150 (33.7)	153 (34.4)	167 (36.5)	176 (39)	189 (41.7)	0.074
3-vessel disease	532 (23.6)	106 (23.8)	105 (23.6)	110 (24.1)	107 (23.7)	104 (23)	0.996
**Location of target lesion**,** n(%)**							
LM	71 ( 3.2)	16 (3.6)	15 (3.4)	14 (3.1)	14 (3.1)	12 (2.6)	0.945
LAD	1861 (82.7)	375 (84.3)	367 (82.5)	375 (82.1)	375 (83.1)	369 (81.5)	0.833
LCX	1094 (48.6)	215 (48.3)	217 (48.8)	218 (47.7)	219 (48.6)	225 (49.7)	0.985
RCA	1112 (49.4)	204 (45.8)	210 (47.2)	237 (51.9)	226 (50.1)	235 (51.9)	0.246
**Characteristics of lesions**,** n(%)**							
Occlusion	297 (13.2)	46 (10.3)	55 (12.4)	74 (16.2)	63 (14)	59 (13)	0.122
CTO	196 ( 8.7)	34 (7.6)	35 (7.9)	41 (9)	35 (7.8)	51 (11.3)	0.259
Ostial lesion	247 (11.0)	60 (13.5)	49 (11)	48 (10.5)	52 (11.5)	38 (8.4)	0.184
Bifurcation lesion	397 (17.6)	96 (21.6)	95 (21.3)	77 (16.8)	63 (14)	66 (14.6)	0.003
Restenosis lesion	29 ( 1.3)	8 (1.8)	4 (0.9)	7 (1.5)	4 (0.9)	6 (1.3)	0.694
total stent length, mm	42.0 (24.0, 66.0)	36.0 (23.0,64.0)	42.0 (24.0, 67.5)	42.5 (27.0, 69.0)	42.5 (24.0, 64.0)	45.0 (28.0, 68.5)	0.048
diameter of stents, mm	3.1 ± 0.9	3.1 ± 0.4	3.1 ± 1.4	3.1 ± 0.4	3.1 ± 0.4	3.1 ± 1.3	0.612

Data are shown as mean ± standard deviation (SD) ) or median (IQR) for continuous variables for continuous variables and proportions (%) for categorical variables

Note: NHHR, non-high-density lipoprotein cholesterol to high-density lipoprotein cholesterol ratio; HF, heart failure, AF, atrial fibrillation; LVEF, left ventricular ejection fraction; STEMI, ST-segment myocardial infarction; NSTE-ACS, non-ST elevation acute coronary syndromes; SA, stable angina; MI, myocardial infarction; TC, total cholesterol; TG, triglyceride; HDL-C, high density lipoprotein cholesterol; LDL-c, low density lipoprotein cholesterol; LM, left main coronary artery; LAD, left anterior descending; LCX, left circumflex artery; RCA, right coronary artery; CTO, chronic total occlusions; *P* values in bold are < 0.05

### Univariable and multivariable logistic regression models to evaluate the association between the NHHR and MACCEs in patients undergoing PCI

The univariable analysis results are provided in Supplement Table 1. Univariable analysis showed that age, higher proportions of history of heart failure, atrial fibrillation, MI, or stroke, greater percentages of 3-vessel disease, CTO, total stent length, and stent diameter were associated with MACCEs. Table 2 demonstrates the relationship between the NHHR and MACCEs using the multivariable logistic regression model analysis. In the crude model (non-adjusted model), the adjusted ORs (95% CIs) for the patients in quintiles 2, 3, 4, and 5 were 0.84 (0.56–1.26), 0.62 (0.41–0.96), 1.00 (0.68–1.48), and 1.10 (0.75–1.61), respectively, (reference group: quintile 1). In model I (adjusted for age and sex), the adjusted ORs (95% CIs) for the patients in quintiles 2, 3, 4, and 5 were 0.85 (0.57–1.28), 0.63 (0.41–0.98), 1.03 (0.701–1.52), and 1.16 (0.79–1.71), respectively (reference group: quintile 1). In Model II (adjusted for age, sex, smoking status, medical history of diseases [MI, heart failure, atrial fibrillation, stroke, hypertension, or diabetes], use of aspirin, statins, or β-blockers, presence of 3-vessel disease, total stent length, and stent diameter), the adjusted ORs (95% CIs) for patients in quintiles 2, 3, 4, and 5 were 0.79 (0.52–1.20), 0.64 (0.42–0.99), 1.00 (0.67–1.48), and 1.17 (0.74–1.64), respectively (reference group: quintile 1).

**Table 2:** Logistic regression analysis results for the association between the NHHR and the risk of developing MACCEs in patients included in this study

NHHR (quintiles )	Number of MACCEs	Crude		Model I		Model II	
		OR (95%CI)	*P*-value	OR (95%CI)	*P*-value	OR (95%CI)	*P*-value
**Q1(< 2.15)**	58	1(Ref)		1(Ref)		1(Ref)	
**Q2(2.15–2.77)**	50	0.84 (0.56,1.26)	0.412	0.85 (0.57,1.28)	0.434	0.79 (0.52,1.20)	0.266
**Q3(2.78–3.39)**	39	0.62 (0.41,0.96)	0.030	0.63 (0.41,0.98)	0.038	0.64 (0.42,0.99)	0.047
**Q4(3.40–4.22)**	59	1.00 (0.68,1.48)	0.983	1.03 (0.70,1.52)	0.887	1.00 (0.67,1.48)	0.984
**Q5(> 4.22)**	64	1.10 (0.75,1.61)	0.632	1.16 (0.79,1.71)	0.448	1.17 (0.74,1.64)	0.623

**Note: Abbreviation**: OR, odds ratio; CI, confidence interval

MACCEs, major adverse cardiac and cerebrovascular events;

MACCEs defined as a composite of cardiac mortality, acute myocardial infarction, stroke, repeat revascularization

**Crude model**: no covariables were adjusted.

**Model I**: adjusted for age, sex.

**Model II**: adjusted for age, sex, smoking status, medical history of diseases (myocardial infarction, heart failure, atrial fibrillation, stroke, hypertension, diabetes), aspirin use, statin use, β-blocker use, 3-vessel disease, total stent length, and diameter of stents.

### U-shaped relationship between the NHHR and MACCEs

**Fig. 2:**
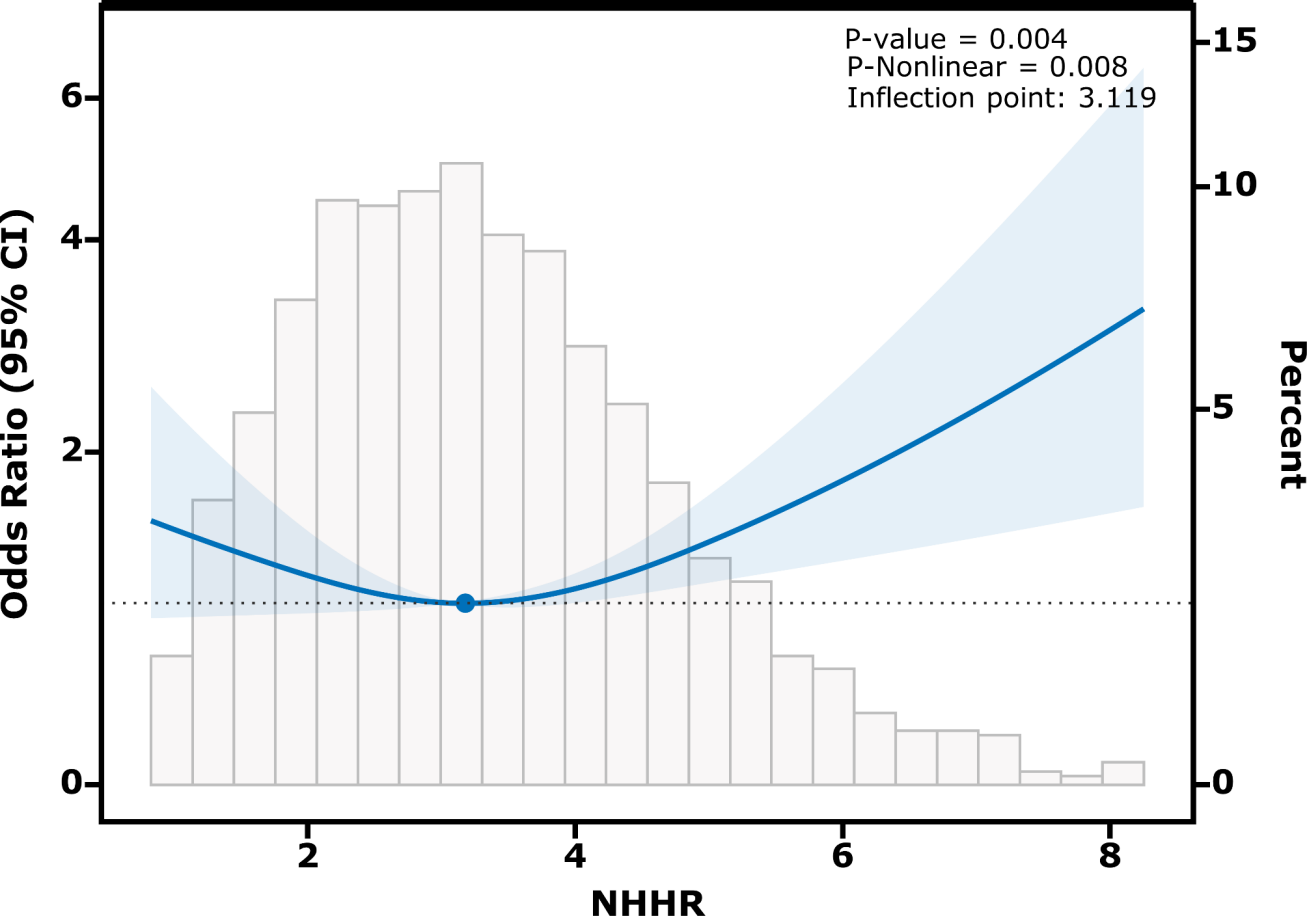
Association between NHHR and MACCEs in CAD patients with PCI. Each Odds ratio was computed with a NHHR level of 3.119 as the reference. Adjusted age, sex, smoking status, medical history of diseases (myocardial infarction, heart failure, atrial fibrillation, stroke, hypertension, diabetes), aspirin use, statin use, β-blocker use, 3-vessel disease, total stent length, and diameter of stents. The solid line and blue area represent the estimated values and their corresponding 95% CIs, respectively (NHHR, non-high-density lipoprotein cholesterol to high-density lipoprotein cholesterol ratio; MACCEs, major adverse cardiac and cerebrovascular events; CAD, coronary artery disease; PCI, percutaneous coronary intervention)

We further explored whether a non-linear association was present between the NHHR and MACCEs in all patients by performing RCS analysis (after adjusting for confounding factors as those in the logistic regression model II)(Fig. 2). A typical binary logistic regression model was also employed to fit the data (Supplemental Table 2). Overall, this analysis concluded that a U-shape association existed between the NHHR and MACCEs. After applying a recursive algorithm, the inflection point was identified at an NHHR of 3.119. In cases where the NHHR was < 3.119, MACCE risk decreased with an adjusted OR of 0.734 (95% CI, 0.551–0.978) for every one unit increment in the NHHR. Conversely, in cases where the NHHR was ≥ 3.119, MACCE risk increased with an adjusted OR of 1.231 (95% CI, 1.038–1.460) for each one unit increment in the NHHR (*P* values for log-likelihood ratio < 0.05) (Table 3).

**Table 3:** Threshold effect analysis of the NHHR on MACCEs

Model	Per-1 unit increase	
	Odds ratios^a^ (95% CI)	*P*- value
**Turning point (K)**	3.119	
**NHHR < K**	0.734 (0.551, 0.978)	0.035
**NHHR ≥ K**	1.231 (1.038, 1.460)	0.017
P for log likelihood ratio test*		0.008

^a^ Odds ratios were derived from multivariable logistic regression analysis

Note: The model was adjusted for age, sex, smoking status, medical history of diseases (myocardial infarction, heart failure, atrial fibrillation, stroke, hypertension, diabetes), aspirin use, statin use, β-blocker use, 3-vessel disease, total stent length, and diameter of stents.

### Subgroup analyses

**Fig. 3:**
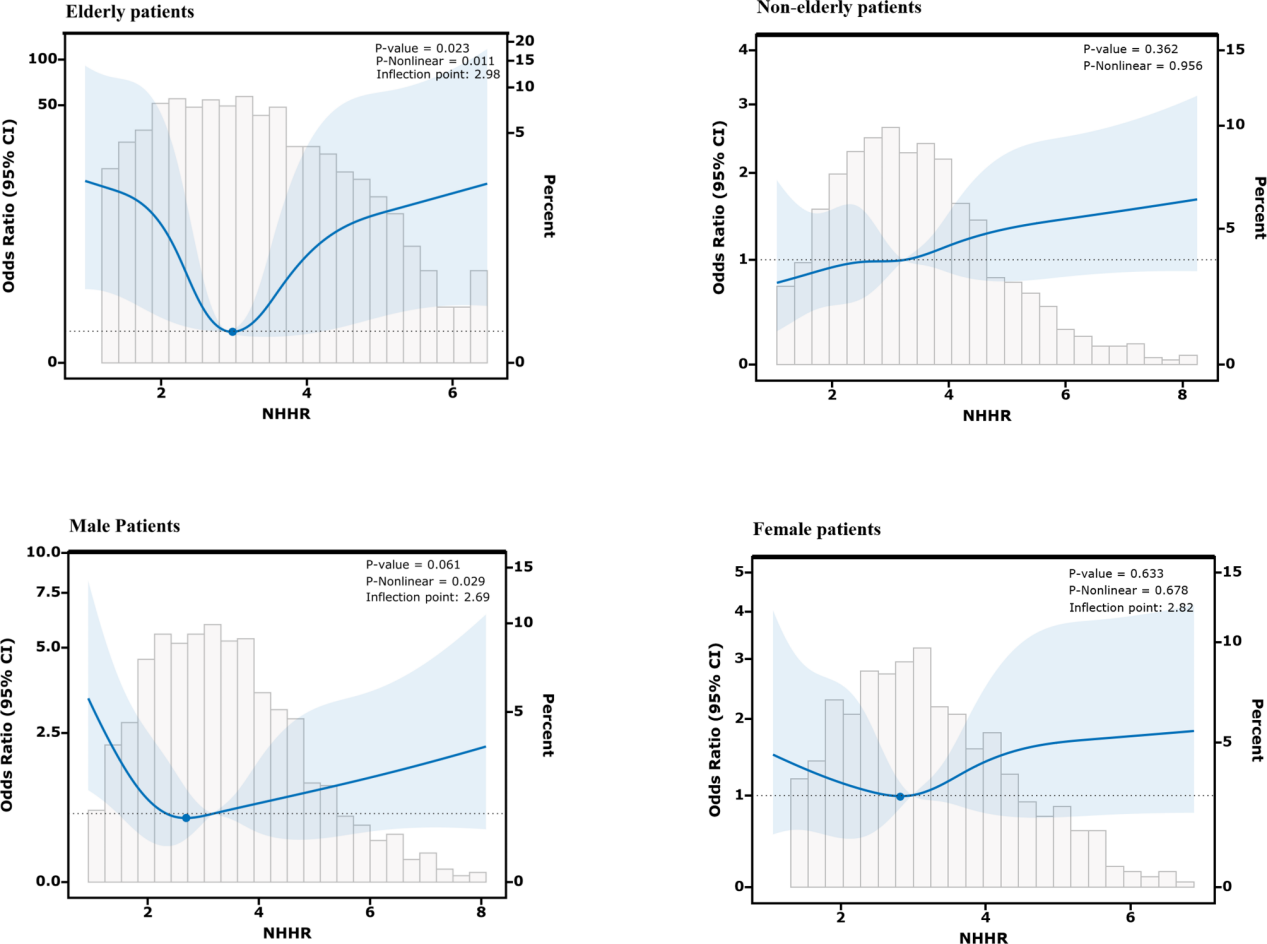
The relationship between the NHHR level and MACCEs. Relationship in the elderly, nonelderly, female, male patients, respectively. Only 95% of the data is displayed. Odds ratios are indicated by solid lines and 95% CIs by shaded areas

Stratified and interaction analyses were conducted to assess the associations between baseline NHHR and MACCE risk in various subgroups, including those of sex, age (< 65 years vs. ≥65 years), clinical presentation (STEMI vs. SA vs. NSTE-ACS), smoking status (no vs. yes), hypertension (no vs. yes), diabetes (no vs. yes), heart failure (no vs. yes), and CTO (no vs. yes), as detailed in Table 4. The U-shaped association was consistent across all subgroups, except for the subgroup of < 65 years of age. In the < 65 years of age subgroup, an increase in the NHHR was associated with a corresponding increase in MACCE risk. The RCS model was further utilized to better visualize the relationship between the NHHR and MACCE risk across different age groups and sexes, demonstrating findings consistent with the subgroup analyses (Fig. 3). Finally, the interaction analysis revealed no significant interactions in the association between baseline NHHR and MACCEs (all P for interaction > 0.05).

**Table 4:** The subgroup analysis of the relationship between NHHR and risk of MACCEs

Subgroups	quintiles of NHHR					*P* for interaction
	Q1	Q2	Q3	Q4	Q5	
	< 2.15	2.15–2.77	2.78–3.39	3.40–4.22	> 4.22	
**Sex**						0.830
**Female**	1(Ref)	0.78 (0.35,1.76)	0.79 (0.35,1.77)	1.41 (0.65,3.05)	1.43 (0.66,3.1)	
**Male**	1(Ref)	0.79 (0.49,1.29)	0.57 (0.34,0.97)	0.9 (0.56,1.44)	1.05 (0.66,1.68)	
**Age**,** years**						0.294
**<65**	1(Ref)	1.02 (0.56,1.80)	1.01 (0.55,1.85)	1.45 (0.83,2.53)	1.47 (0.85,2.55)	
**≥ 65**	1(Ref)	0.64 (0.35,1.17)	0.40 (0.20,0.78)	0.67 (0.36,1.26)	0.92 (0.49,1.73)	
**Clinical presentation**						0.690
**STEMI**	1(Ref)	1.42 (0.61,3.27)	0.56 (0.22,1.41)	1.03 (0.44,2.41)	1.25 (0.53,2.93)	
**SA**	1(Ref)	0.53 (0.18,1.56)	0.49 (0.15,1.58)	0.96 (0.34,2.66)	1.05 (0.36,3.06)	
**NSTE-ACS**	1(Ref)	0.67 (0.38,1.18)	0.71 (0.41,1.25)	1.02 (0.61,1.72)	1.16 (0.69,1.94)	
**Smoking status**						0.934
**No**	1(Ref)	0.8 (0.48,1.32)	0.63 (0.37,1.07)	1.08 (0.66,1.75)	1.23 (0.76,1.99)	
**Yes**	1(Ref)	0.83 (0.40,1.74)	0.63 (0.29,1.37)	0.85 (0.42,1.74)	0.99 (0.49,2.00)	
**Hypertension**						0.635
**No**	1(Ref)	0.84 (0.46,1.51)	0.55 (0.29,1.05)	1.21 (0.69,2.13)	1.07 (0.59,1.93)	
**Yes**	1(Ref)	0.77 (0.43,1.39)	0.72 (0.39,1.31)	0.87 (0.49,1.55)	1.21 (0.70,2.09)	
**Diabetes**						0.858
**No**	1(Ref)	0.72 (0.45,1.15)	0.62 (0.38,1.02)	0.98 (0.63,1.54)	1.14 (0.73,1.79)	
**Yes**	1(Ref)	1.33 (0.52,3.36)	0.82 (0.31,2.13)	1.02 (0.41,2.53)	1.35 (0.56,3.25)	
**Heart failure**						0.576
**No**	1(Ref)	0.71 (0.45,1.13)	0.59 (0.37,0.95)	1.02 (0.67,1.56)	1.08 (0.71,1.65)	
**Yes**	1(Ref)	1.34 (0.47,3.81)	1.01 (0.34,3.04)	0.86 (0.26,2.83)	1.56 (0.51,4.77)	
**CTO**						0.133
**No**	1(Ref)	0.91 (0.58,1.43)	0.79 (0.50,1.26)	1.17 (0.76,1.82)	1.33 (0.86,2.06)	
**Yes**	1(Ref)	0.40 (0.13,1.27)	0.11 (0.03,0.46)	0.44 (0.14,1.34)	0.54 (0.16,1.43)	

Note: The model was adjusted, if not stratified, for age, sex, smoking status, medical history of diseases (myocardial infarction, heart failure, atrial fibrillation, stroke, hypertension, diabetes), aspirin use, statin use, β-blocker use, 3-vessel disease, total stent length, and diameter of stents

## Discussion

In this cohort study, patients with CAD undergoing PCI were followed up for a mean duration of 29.8 months, and the results showed a U-shaped relationship between baseline NHHR and MACCE incidence, with the inflection point at an NHHR of approximately 3.119 and the minimal risk at NHHR values ranging from 2.78 to 3.39. Moreover, the magnitude of these associations is clinically important, particularly among those with extremely high and low NHHR values.

The NHHR is proposed as a novel comprehensive lipid index that incorporates all relevant information on pro-atherosclerotic and anti-atherosclerotic lipoprotein particles, thereby reflecting the balance between the various lipoproteins [26, 27]. Previous studies have suggested that the NHHR substantially outperforms traditional lipid parameters in evaluating atherosclerosis [8]. Furthermore, the NHHR has been demonstrated to have superior predictive capabilities for metabolic conditions such as diabetes, metabolic syndrome, and insulin resistance that exceed the predictive value of individual lipid markers, including LDL-C, non-HDL-C, and HDL-C [8, 15, 28]. Prior research has also reported the association between the NHHR and CAD risk [17, 18]. Although the relationship between the NHHR and CAD risk has been established, only one study has investigated the relationship between the NHHR and MACCEs in patients with CAD. Furthermore, the optimal NHHR value has not been well defined in patients with CAD. A study by Jiayin You et al. [18]has found that baseline NHHR values were associated with MACCEs. However, the researchers failed to provide a comprehensive understanding of the exact relationship between the NHHR and MACCE risk because they conducted a subgroup analysis with only MACCEs as the stratification factor, with no adjustments for potential confounding factors or evaluation of dose–response relationships. Consequently, their conclusions were constrained, underscoring the need for the current investigation.

In biomedical research, the relationship between exposures and outcomes is known to exhibit non-linear patterns. Therefore, researchers require a better method to analyze the dose–response relationship between the NHHR and MACCE risk in patients with CAD undergoing PCI, along with adjusting for various covariables and conducting subgroup analyses. The current study revealed that the NHHR was significantly associated with MACCEs, with effective adjustment for potential confounders further improving the reliability of our results. Additionally, we extensively employed RCS to reveal a U-shaped association between the NHHR and MACCE incidence risk. Moreover, this study used a two-piecewise linear regression model to determine the MACCE incidence with an inflection point at an NHHR of approximately 3.119 and minimal risk at NHHR values ranging from 2.78 to 3.39. All these findings were partially consistent with the previous findings by a UK Biobank study, wherein a non-linear association was observed between lipids, lipoproteins, and fatal cardiovascular disease [29].

In the present study, sex, age (< 65 years vs. ≥65 years), smoking status, hypertension, diabetes, heart failure, and CTO were utilized as stratification variables. The results indicated no significant interactions between the NHHR and MACCEs across any subgroup. Nevertheless, a U-shaped association between the NHHR and MACCEs was consistently observed across all subgroups, except in the subgroup of ≤ 65 years of age. In individuals < 65 years of age, extremely low NHHR values were not associated with a significant increase in MACCE risk. Furthermore, this study detected an inconsistent relationship between the NHHR and MACCE risk in patients of < 65 and ≥ 65 years of age who were undergoing PCI. Hence, we further explored this age-related phenomenon and noted that the increase in MACCE risk with escalating NHHR in patients of < 65 years of age who were undergoing PCI may be attributed to several factors. For example, younger patients typically exhibit more active lipid metabolism, wherein reactive oxygen species can damage endothelial cells and exacerbate atherosclerosis [30]. Additionally, younger patients generally have fewer comorbidities and lesser cumulative vascular damage than their older counterparts. Studies on lipid management also frequently highlight that younger individuals tend to experience more benefits from aggressive lipid control than older adults [31]. In contrast, older patients often have relatively more complex clinical presentations, comprising multiple comorbidities and long-term vascular diseases such as hypertension, obesity, dementia, and diabetes. All these conditions can influence the response of the older population to lipid dysregulation. Previous studies have also reported an association between lower LDL-C levels and a higher risk of adverse events, including hemorrhagic stroke and dementia [32]. This finding may be explained by the observed differences between older and younger individuals. However, the exact mechanisms of these age-related changes require further investigation.

This study offers novel insights into the relationship between the NHHR and MACCEs in patients with CAD undergoing PCI. Specifically, among patients with an NHHR of < 3.119, MACCE risk significantly decreased with the NHHR. Moreover, the lower NHHR values were attributed to higher HDL-C levels. Previous studies have further shown that excessively high HDL-C levels paradoxically lead to heightened senescence and impaired endothelial function, thereby diminishing its protective effect [33]. Current evidence indicates that heightened HDL-C concentrations may result in an elevation in cholesterol-overloaded HDL particles, which may be less effective in preventing atherosclerosis development [34, 35]. We hypothesize that such alterations in the conformational and functional properties of HDL particles may underlie the negative association between the NHHR and MACCEs, potentially leading to adverse effects. Furthermore, MACCE risk significantly increased with the NHHR value in patients with an NHHR of ≥ 3.119. Higher NHHR values correspond to elevated non-HDL-C and diminished HDL-C levels, potentially leading to coronary inflammation and heightened rupture risk of coronary plaques via mechanisms such as oxidative stress and inflammatory processes. However, additional research is necessary to validate these results.

### Strengths and limitations of the study

This study demonstrates a few noteworthy strengths, including employing RCS to comprehensively assess potential relationships and enhance our study’s ability to uncover the true associations between exposure and outcome. The present study also examined the reliability of the results across different populations via subgroup analysis. Additionally, we used real-world data to design this large-scale population study. However, this study has several limitations that should be considered. First, the study population consisted of only Chinese patients with CAD who were undergoing PCI, which may limit the generalizability of our findings to other populations. Second, the lack of time-related data during follow-up restricted us from applying Cox regression analysis to investigate the relationship between the NHHR and MACCEs, which might weaken the results. Thirdly, the original database does not include evaluation of completeness of revascularization, which is independently associated with MACCEs. And this may affect the clinical significance of NHHR. Therefore, further large-scale cohort studies in diverse populations are warranted to validate the applicability of our conclusions.

## Conclusion

Our study revealed a U-shaped association between the NHHR and MACCE occurrence in Chinese patients hospitalized with CAD who were undergoing PCI, with lower and higher values of the NHHR being associated with an increased risk of MACCE development. Our findings underscore that the NHHR may serve as a valuable lipid index to assess MACCEs in this patient population.

## Electronic supplementary material

Below is the link to the electronic supplementary material.

Supplementary Material 1

Supplementary Material 2

Supplementary Material 3

Supplementary Material 4

## Data Availability

No datasets were generated or analysed during the current study.
