# Supplementary material for: Association between the non-high-density lipoprotein cholesterol to high-density lipoprotein cholesterol ratio (NHHR) and cardiovascular outcomes in patients undergoing percutaneous coronary intervention: a retrospective study
Source: Lipids Health Dis. 2024 Oct 1;23:324. doi: 10.1186/s12944-024-02309-4 (PMC11443751; doi:10.1186/s12944-024-02309-4)
Supplement: Supplementary file 2 — Supplementary Material 2 [file 12944_2024_2309_MOESM2_ESM.pdf]

Bullet Edits

EDITORIAL CERTIFICATE

This document certifies that the paper listed below was edited and proofread for proper English language, grammar, punctuation, spelling, and overall style by one or more than one highly qualified native speakers at Bullet Edits. All of the suggested amendments were tracked with the Microsoft Word "Track Changes" feature. Therefore, the author had the option to reject or accept each change individually.

Manuscript Title:

Association between the non-high-density lipoprotein cholesterol to high-density lipoprotein cholesterol ratio (NHHR) and cardiovascular outcomes in patients undergoing percutaneous coronary intervention：a retrospective study

Anti-counterfeiting Code:

50bbe3d433dc3f942687c0da83ed3135

Date Issued:

2024-08-09 21:43:10

Bullet Edits is a registered company headquartered in the UK with a global presence. We offer a range of editing, proofreading services to authors. Our Ph.D. editors are all native English speakers from the USA and UK. Authors who work with Bullet Edits are guaranteed excellent language quality and timely delivery.

BULLET EDITS LIMITED ★ COMPANY NO. 13345261 ★ SEAL ★ ENGLAND AND WALES

Address: BULLET EDITS LIMITED, 85 Great Portland Street, London,UK
Tel: UK. (+44)20457 70286 / US.(+1)312-313-9179
Web: www.bulletedits.cn/Email: info@bulletedits.cn
VAT: GB 378 9316 43
