# Supplementary material for: Association between the non-high-density lipoprotein cholesterol to high-density lipoprotein cholesterol ratio (NHHR) and cardiovascular outcomes in patients undergoing percutaneous coronary intervention: a retrospective study
Source: Lipids Health Dis. 2024 Oct 1;23:324. doi: 10.1186/s12944-024-02309-4 (PMC11443751; doi:10.1186/s12944-024-02309-4)
Supplement: Supplementary file 3 — Supplementary Material 3 [file 12944_2024_2309_MOESM3_ESM.pdf]

20240811214006102012662524768665

WORD COUNT	3441	TIME SUBMITTED	11-AUG-2024 09:40PM
		PAPER ID	111003053

Title Page

Association between the non-high-density lipoprotein cholesterol to high-density lipoprotein cholesterol ratio (NHHR) and cardiovascular outcomes in patients undergoing percutaneous coronary intervention： a retrospective study

Abstract

Background: Dyslipidemia was significantly related to adverse outcomes in patients with coronary artery disease (CAD). Non-high-density lipoprotein cholesterol (non-HDL-C) to high-density lipoprotein cholesterol (HDL-C) ratio (NHHR) is a novel comprehensive lipid index. However, limited evidence supports the relationship of NHHR with the likelihood of developing detrimental outcomes in patients with CAD. The purpose of this study was to examine any links between NHHR levels and adverse outcomes in CAD patients who had undergone percutaneous coronary intervention (PCI).

Methods: Among 2253 CAD patients receiving PCI, 2251 with both total cholesterol (TC) and HDL-C were analyzed. All participants were classified into 5 groups by quintiles of NHHR levels. The primary outcome was specified as major adverse cardiovascular and cerebrovascular events (MACCEs), comprising cardiac mortality, acute myocardial infarction (AMI), stroke, and repeat revascularization. Multivariable logical regression analysis was utilized to determine the relationship NHHR and MACCEs. Restricted cubic spline (RCS) was used to quantify nonlinearity. In order to confirm the consistency of these associations, subgroup and interaction analyses were conducted.

Results Over a median follow-up span of 29.8 months, 270 individuals experienced MACCEs. Following adjustment for confounding variables, patients in the bottom (Q1) and top (Q5) quintile of NHHR were correlated with a higher risk of MACCEs (adjusted OR, 1.61 [95% CI, 1.03 to 2.52] and adjusted OR, 1.79 [95% CI, 1.19 to 2.78], respectively), compared with those in the reference group (Q3: 2.78 to 3.39). Moreover, RCS analysis revealed a U-shaped correlation between NHHR and MACCEs, with a two-piecewise regression model pinpointing an inflection at 3.119. This pattern remained consistent across various subgroups, and significant interactions were absent. For the left and right

inflection points, the ORs and 95% CIs were 0.734 (0.551 to 0.978) and 1.231 (1.038 to 1.460), respectively.

Conclusions This research identified a U-shaped link between initial NHHR and the occurrence of MACCEs in CAD patients undergoing PCI. Further, this study also found that more stringent lipid control targets may be necessary for CAD patients undergoing PCI at higher risk for MACCEs, particularly those aged 65 years or younger.

Keywords: Coronary artery disease, NHHR, Major adverse cardiovascular and cerebrovascular events

Introduction

Coronary artery disease (CAD) is a prevalent condition that presents a significant threat to human health, thought to cause 9 million deaths annually [1]. The adoption of reperfusion strategies and enhancement of regional coordinated treatment systems have significantly reduced acute-phase mortality in patients with CAD [2-4]. Despite these advances, the incidence of major adverse cardiovascular and cerebrovascular events (MACCEs) subsequent to percutaneous coronary intervention (PCI) is on the rise [5, 6]. Dyslipidemia is prevalent among patients with confirmed CAD and is associated with adverse outcomes. Identifying residual risk factors in CAD patients undergoing PCI is critical for reducing the risks of MACCEs.

Traditionally, low-density lipoprotein cholesterol (LDL-C) was the main focus of dyslipidemia management in patients with CAD. Despite aggressive LDL-C lowering treatment, patients still face a heightened risk of residual cardiovascular events. The non-high-density lipoprotein cholesterol to high-density lipoprotein cholesterol ratio (NHHR) is a novel comprehensive lipid index, which integrates information on all atherogenic and anti-atherogenic lipid measurements[7]. In clinical practice, the NHHR can be easily obtained from normal lipid profiles at no additional cost[8]. Earlier study has indicated that NHHR [illegible] a significant risk factor for conditions such as insulin resistance, nonalcoholic fatty liver disease[9], carotid atherosclerosis[10-12], diabetes mellitus (DM)[13, 14], hyperglycemia[15] and CAD[16, 17]. Additionally, a study by Jiayin You and colleagues found that baseline NHHR levels were associated

with the progression of CAD and correlated with MACCEs. However, the authors only performed a subgroup analysis using MACCEs as a stratification factor, without testing for potential confounding factors or dose-response relationships. This limitation restricts a thorough investigation of the genuine interrelation between NHHR and the threat of MACCEs. Consequently, the findings of You et al. were limited, necessitating our present study.

This study, therefore, aims to appraise the correlation between baseline NHHR and the risk of MACCEs. The hypothesis of this study posits a potential U-shaped relationship, suggesting the existence of an optimal NHHR range associated with the lowest risk of MACCEs in CAD patients undergoing PCI through a secondary data analysis utilizing existing data from a published source[18].

Methods

Study design and participants

The data is from the "DATADRYAD" database (www.Datadryad.org), which provides free access to raw data downloads. Compliance with Dryad's Terms of Service was ensured, and the relevant data packages were appropriately cited in the study: Yao H M, Wan Y D, Zhang X J, Shen D L, Zhang J Y, Li L, Zhao L S, Sun T W. (2014). Long-term follow-up results in patients undergoing percutaneous coronary intervention (PCI) with drug-eluting stents: Results from a single high-volume PCI center. Conducted in the interim of July 2009 and August 2011 at the First Affiliated Hospital of Zhengzhou University, a high-volume PCI center in China, this study involved 2533 participants who underwent PCI using standard techniques. Prior to the coronary intervention, all patients were given a loading dose of 300 mg of aspirin and 300 mg of clopidogrel, unless they were already on antiplatelet therapy. Post-PCI, patients followed a standard dual antiplatelet regimen, consisting of 100 mg of aspirin and 75 mg of clopidogrel daily for at least one year. The participants were monitored for a median of 29.8 months, with interquartile ranges from 25.6 to 34 months. After excluding incomplete and missing data on total

cholesterol (TC) and high-density lipoprotein cholesterol (HDL-C), the study included data from 2251 patients (see Fig. 1).

Ethics approval and consent to participate

The research protocol received approval from the Ethics Committee of the First Affiliated Hospital of Zhengzhou University. Given the retrospective design of this research, the committee waived the need for informed consent. Helsinki Declaration principles were adhered to in this investigation. Additional details can be found in a prior investigation. No further ethical declaration was necessary for this study, as the dataset's public policy statement had already been sanctioned by the ethics committee.

Data collection and outcome definition

Upon admission, data such as age, sex, and smoking status were recorded. Clinical presentations, including ST-segment myocardial infarction (STEMI), non-ST elevation acute coronary syndromes (NSTE-ACS), and stable angina (SA), were noted along with comorbidities such as heart failure, atrial fibrillation, prior myocardial infarction (MI), prior stroke, hypertension, and diabetes mellitus (DM). Angiographic information included the surgical techniques employed, the specific locations of target lesions in the coronary arteries (left main coronary artery [LM], left circumflex artery [LCX], left anterior descending artery [LAD], and right coronary artery [RCA]), and the number of vessels involved. Lesion characteristics including whether they were occluded, presented as chronic total occlusions (CTO), were located at the ostium, or were bifurcation lesions were recorded. Furthermore, information regarding the number of vessels that underwent treatment and the number of stents used, including their length and diameter, were documented. Data regarding various drugs including aspirin, clopidogrel, β-blockers, and statins were collected. Follow-ups with the patients were conducted through outpatient visits, readmissions, or telephone communication. Hypertension was identified based on the use of antihypertensive medications or a self-reported history of elevated blood pressure. DM history was determined by the use of antidiabetic medications or self-reported DM. Individuals who had smoked within the last decade were classified as smokers.

Patients' medical records were used to source laboratory test results. Fasting blood samples underwent standard tests to determine levels of creatinine, glycemia, triglycerides (TG), total cholesterol (TC), low-density lipoprotein cholesterol (LDL-C), and high-density lipoprotein cholesterol (HDL-C). Non-HDL-C was computed by subtracting HDL-C from TC. The NHHR was derived by calculating the ratio between non-HDL-C and HDL-C.

Outcome Measures

The outcome of this study was the incidence of MACCEs during follow-up. MACCEs incidence was identified as comprising cardiac mortality, acute myocardial infarction (AMI), stroke, and repeat revascularization. All participants were followed up until cardiac death, AMI, stroke, repeat revascularization, or the censoring date (August 2011), whichever came first. Clinical follow-up was was carried out phone interviews, patient visits, and a retrospective examination of medical records. Data entry was performed by independent researchers, and a separate committee adjudicated clinical events.

Statistical analysis

Participants were categorized into quintiles in terms of NHHR levels: quintile (Q)1 (n = 445, NHHR <2.15), Q2 (n = 445, $2.15 \leq$ NHHR ≤ 2.77), Q3 (n = 357, $2.78 \leq$ NHHR ≤ 3.39), Q4 (n = 353, $3.40 \leq$ NHHR ≤ 4.22), and Q5 (n = 392, NHHR > 4.22), and as stated in the previous studies[19, 20]. The chi-square test evaluated categorical variables, represented as numbers (n) and percentages (%). Continuous variables were presented as mean ± standard deviation if they followed a normal distribution, and as median (interquartile range) if they had a non-normal distribution. One-way analysis of variance (ANOVA) was employed for normally distributed variables, while the Kruskal-Wallis test analyzed those with non-normal distribution.

The NHHR–MACCEs correlation was tested using both univariate and multivariate logistic regression models. Initially, the crude model had no covariates adjusted. Model I was adjusted for sex and age. Model II included further adjustments for sex, age, smoking status, medical history (including

MI, heart failure, atrial fibrillation, stroke, hypertension, DM), aspirin use, statin use, β-blocker use, 3-vessel disease, total stent length, and stent diameter. These adjustments were based on their associations with outcomes, clinical relevance, literature reports, and a change in matched odds ratio of at least 10%[21, 22]. Generalized Additive Models (GAM) depicted the nonlinear relationship between NHHR and MACCEs and were utilized to analyze the threshold saturation effect. A two-piecewise linear regression model was applied to identify the NHHR threshold saturation effect on MACCEs risk, when a non-linear relationship was noted [23]. The inflection point on the smoothing curve was pinpointed automatically by the recursive method which utilized the maximum model likelihood. The log-likelihood ratio test was made use of to identify the most suitable model describing the NHHR-MACCEs relationship. Additionally, the bootstrap resampling technique was applied to compute the 95% confidence interval for the inflection point, as elaborated in previous analyses [24].

Stratified analyses were applied to evaluate the heterogeneities in the associations between baseline NHHR levels and MACCEs across different subgroups, including MACCEs risk group, sex, age (<65 years vs. ≥65 years), smoking status (no vs. yes), presence of CTO (no vs. yes), and medical history of diabetes, hypertension, and heart failure. Data analyses were carried out using the statistical software packages R and EmpowerStats. The findings are reported as ORs with corresponding 95%CIs. Statistical significance was considered for p-values < 0.05.

Results

Baseline characteristics of the study participants

Out of the 2533 CAD participants who underwent PCI, 282 were excluded from this study. Among the excluded individuals, 4 had confusing data and 278 had missing data (272 for TC and 6 for HDL-C), leaving 2251 participants for analysis. Figure 1 illustrates this study flowchart. The mean (SD) age of the study population was 60.0 (11.1) years, with 718 (31.9%) men. Table 1 describes the baseline characteristics of the population according to NHHR quintiles. Patients in the highest NHHR quintile were more likely to be smokers, have elevated levels of TC, TG, and LDL-C, higher rates of STEMI,

and a greater history of DM. Conversely, patients in the lowest NHHR quintile were inclined to be older, of the male sex, have NSTE-ACS, with higher HDL-C levels, along with a higher percentage of bifurcation lesions (all $P < 0.05$).

Univariate and Multivariable logistic regression models evaluating the correlation between NHHR and MACCEs in patients receiving PCI.

The results of the univariate analysis are shown in **Supplement Table 1**. The results of the univariate analysis showed that age, higher proportions of history of heart failure, atrial fibrillation, prior myocardial infarction, higher percentages of 3-vessel disease, CTO, total stent length, and diameter of stents were correlated with MACCEs. Subsequently, when NHHR levels were classified into quintiles, individuals in Q3 exhibited the lowest risk of MACCEs (see **Table 2**). This study employed multivariable logistic regression models in order to assess the relationships linking NHHR and MACCEs, as shown in Table 2. Analyzing NHHR as a continuous variable revealed no statistically significant association with MACCEs across all three models (all $P > 0.05$). Further exploration considered NHHR level Q3 (3.40-4.22) as the reference. In the crude model (no adjustments), the adjusted ORs (95% CIs) for individuals in Q1, Q2, Q4, and Q5 were 1.61 (1.05 to 2.47), 1.36 (0.87 to 2.11), 1.39 (0.89 to 2.18), and 1.76 (1.16 to 2.69), respectively, compared to Q3 (p for trend = 0.399). In Model 2, adjusted for age and sex, the adjusted ORs (95% CIs) for Q1, Q2, Q4, and Q5 were 1.57 (1.02 to 2.43), 1.34 (0.86 to 2.08), 1.52 (0.91 to 2.19), and 1.78 (1.17 to 2.77), respectively, compared to Q3 (p for trend = 0.275). In Model 3, adjusted for age, sex, smoking status, medical history (including MI, heart failure, atrial fibrillation, stroke, hypertension, DM), aspirin use, statin use, β-blocker use, 3-vessel disease, total stent length, and stent

diameter, the adjusted ORs (95% CIs) for Q1, Q2, Q4, and Q5 were 1.61 (1.03 to 2.52), 1.27 (0.81 to 1.98), 1.59 (1.03 to 2.23), and 1.79 (1.19 to 2.78), respectively, compared to Q3 (p for trend = 0.252). These findings suggest a non-linear relationship between NHHR and MACCEs (**Fig 2**).

U-shaped relationship between the NHHR and MACCEs

This study utilized RCS and the two-piecewise logistic regression model to further examine the association between NHHR and MACCEs. The findings indicated that the association between NHHR and MACCEs followed a U-shaped pattern. Using a recursive algorithm, we identified the inflection point for MACCEs at 3.119. According to the two-piecewise logistic regression models, for every 1 unit increase in NHHR, there was a 23.6% decrease in MACCEs (adjusted OR, 0.734; 95% CI, 0.551 to 0.978) among participants with NHHR below 3.119, and a 23.1% increase in MACCEs (adjusted OR, 1.231; 95% CI, 1.038 to 1.460) among those with NHHR at or above 3.119 (P values for log-likelihood ratio < 0.05) (Table 3).

The results of subgroup analyses

Stratified analyses were then conducted, stratifying by sex, age (<65 years vs ≥65 years), smoking status (no vs yes), hypertension (no vs yes), DM (no vs yes), heart failure (no vs yes), and CTO (no vs yes) as shown in **Table 4.** The U-shaped association between NHHR and MACCEs was consistently observed across all subgroups except those aged < 65 years (all *P* for interaction > 0.05). A RCS model revealed an approximate linear relationship in the nonelderly (<65 years) subgroup (P for non-linearity=0.956) (**Fig 3**).

Discussion

In this cohort study of CAD patients receiving PCI, with a mean monitoring period of 29.8 months, a U-shaped relationship was observed linking the baseline NHHR and the incidence of MACCEs. The inflection point was identified at approximately 3.119, with the lowest risk occurring at NHHR levels between 2.78 and 3.39. The magnitude of the associations is clinically important, especially among those

with very high and low NHHR levels. Furthermore, this study found that more stringent lipid control targets may be necessary for CAD patients undergoing PCI at higher risk for MACCEs, particularly those aged 65 years or younger.

The NHHR is proposed as a novel, comprehensive lipid index that integrates all relevant information about atherosclerosis-inducing and atherosclerosis-inhibiting lipoprotein particles, thereby reflecting the equilibrium among various lipoproteins [25, 26]. Research has suggested that NHHR remarkably outperforms conventional lipid parameters in assessing atherosclerosis [7]. Additionally, in the context of metabolic disorders, prior research has reported that NHHR has superior prognostic capabilities for conditions such as diabetes, metabolic syndrome, and insulin resistance, surpassing the predictive value of individual lipid markers like LDL-C, HDL-C, and non-HDL-C. Recent studies have shown that NHHR is associated with the risk of CAD[16, 17]. Although the link between NHHR and CAD risk is well-established, only one study has examined the relationship between NHHR and MACCEs in CAD patients. The optimal NHHR target for these patients remains unclear. Research by You et al. [17] indicated that a NHHR were correlated with MACCEs. However, the authors only conducted a subgroup analysis using MACCEs as a stratification factor, without adjusting for potential confounding factors or evaluating dose-response relationships. This limitation hinders a comprehensive understanding of the true relationship between NHHR and MACCEs risk. Consequently, their conclusions were constrained, underscoring the need for our current investigation.

In the field of biomedical research, it is recognized that the relationship between exposures and outcomes may exhibit non-linear patterns. Consequently, researchers need a better method to analyze the dose-response relationship between NHHR and the risk of MACCEs in CAD patients undergoing PCI, while considering various known covariates and conducting subgroup analyses. This investigation identified a remarkable association between NHHR and MACCEs, with effective adjustment for potential confounders enhancing the reliability of the findings. Additionally, extensive use of GAM analysis revealed a U-shaped correlation between NHHR levels and the threat of MACCEs. Furthermore,

this study found the incidence of MACCEs with an inflection point at approximately 3.119 and minimal risk at 2.78 to 3.39 of NHHR level by a two-piecewise linear regression model. These findings align partially with previous results from the UK Biobank, which identified a non-linear relationship between lipids, lipoproteins, and fatal cardiovascular disease [28]. Additionally, this study discovered an incidence of MACCEs with an inflection point around 3.119 and minimal risk at NHHR levels between 2.78 and 3.39, as determined by a two-piecewise linear regression model.

In this study, stratification variables included sex, age (<65 years vs. ≥65 years), smoking status, hypertension, DM, heart failure, and CTO. No significant interaction between NHHR and MACCEs was found in any subgroup. A U-shaped association between NHHR and MACCEs was consistently observed across all subgroups, except for those under 65 years of age. In individuals under the age of 65, extremely low NHHR levels did not exhibit a significantly increased risk of MACCEs. This observation indicates that extremely low NHHR levels appear to be safe for Chinese patients aged 65 years or younger with CAD who have undergone PCI. These findings suggest that more stringent lipid control targets may be necessary for CAD patients undergoing PCI at higher risk for MACCEs, particularly those aged 65 years or younger.

This research offers novel insights into the relationship between NHHR and MACCEs. Specifically, first, among patients with NHHR <3.119, the risk of MACCEs significantly decreased with the NHHR level. Notably, the lower NHHR was caused by higher HDL-C levels. In previous study, excessively high levels of HDL-C can paradoxically led to an increase in senescence and impairment endothelial function, thereby diminishing its protective effect [29]. Current evidence suggests that elevated HDL-C may lead to an accumulation of cholesterol-overloaded HDL particles, which might be less effective in preventing atherosclerosis [30, 31]. Additionally, research has found that changes in the conformational and functional properties of HDL particles may explain the negative association between NHHR and MACCEs, potentially leading to adverse outcomes. Second, the risk of MACCEs significantly increased with the NHHR in patients with an NHHR level of 3.119 or greater. A higher NHHR was associated with

increased non-HDL-C levels and a reduction in HDL-C levels, which could potentially lead to coronary inflammation and a greater susceptibility of coronary plaques to rupture. These effects are likely mediated by oxidative stress and inflammatory processes. It is important to note that additional research is needed to validate our results.

Strengths and limitations

This study demonstrates significant strengths by employing the GAM to elucidate nonlinear relationships. GAM offers distinct advantages in addressing nonlinearity, accommodating non-parametric smoothing, and fitting regression splines to the data. Utilizing GAM enhances our ability to uncover the true associations between exposure and outcome. However, this study has a number of limitations. Firstly, the population studied consists solely of Chinese CAD patients undergoing PCI, which may limit the generalizability of our findings to other populations. Second, the lack of follow-up time data restricted us from using logistic regression analysis to investigate the relationship between NHHR and MACCEs. These factors could potentially weaken the results. Further large-scale cohort studies in diverse populations are necessary to validate the applicability of our conclusions.

Conclusion

This study suggested a U-shaped relationship between NHHR and the occurrence of MACCEs in Chinese hospitalized CAD patients undergoing PCI, indicating that both lower and higher NHHR were linked to an increased risk of developing MACCEs. Additionally, it was found that stricter lipid control targets might be required for CAD patients undergoing PCI who are at higher risk for MACCEs, especially those aged 65 years or younger. These findings emphasize the significance of NHHR in the development of MACCEs.

20240811214006102012662524768665

ORIGINALITY REPORT

9 %

SIMILARITY INDEX

PRIMARY SOURCES

1	www.frontiersin.org<br>Internet	48 words — 1 %
2	lipidworld.biomedcentral.com<br>Internet	44 words — 1 %
3	www.ncbi.nlm.nih.gov<br>Internet	43 words — 1 %
4	www.researchsquare.com<br>Internet	38 words — 1 %
5	www.science.gov<br>Internet	38 words — 1 %
6	assets.researchsquare.com<br>Internet	31 words — 1 %
7	Su Zou, Yingjia Xu. "Association of the triglyceride-glucose index and major adverse cardiac and cerebrovascular events in female patients undergoing percutaneous coronary intervention with drug-eluting stents: A retrospective study", Diabetes Research and Clinical Practice, 2021<br>Crossref	26 words — 1 %
8	Heng Liu, Yu Zhou, Mingchu Jin, Haidong Hao, Yutang Yuan, Hongtao Jia. "The association between	25 words — 1 %

non-high-density lipoprotein cholesterol to high-density lipoprotein cholesterol ratio (NHHR) and prevalence of urinary stones in US adults: a cross-sectional NHANES study", International Urology and Nephrology, 2024
Crossref

9	datadryad.org<br>Internet	18 words — < 1%
10	bmcgastroenterol.biomedcentral.com<br>Internet	18 words — < 1%
11	cisejournal.org<br>Internet	17 words — < 1%
12	portlandpress.com<br>Internet	17 words — < 1%
13	"Sunday, 3 September 2006", European Heart Journal, 08/02/2006<br>Crossref	16 words — < 1%
14	www.nhlbi.nih.gov<br>Internet	16 words — < 1%
15	Xiaoyu Wang, Cheng Huang, Yufei Liu, Yong Han, Haofei Hu. "Association of estimated glomerular filtration rate and incident pre-diabetes: A secondary 5-year longitudinal cohort study in Chinese people", Frontiers in Endocrinology, 2022<br>Crossref	16 words — < 1%
16	Changchun Cao, Cuimei Wei, Yong Han, Jiao Luo, Ping Xi, Jingying Chen, Xiaohua Xiao, Haofei Hu, Dongli Qi. "Association between excessive alcohol consumption and incident diabetes mellitus among Japanese based on propensity score matching", Scientific Reports, 2024<br>Crossref	16 words — < 1%

17 Chun-Chao Huang, Hsin-Fan Chiang, Cheng-Chih Hsieh, Hui-Chen Lin et al. "Percutaneous transluminal angioplasty and stenting of post-irradiation stenosis of the vertebral artery", Journal of Neuroradiology, 2023 — 16 words — < 1%
Crossref

18 Yang Cheng, Zhen Fang, Xinxin Zhang, Yuchen Wen, Jiaqi Lu, Shenghu He, Bing Xu. "Association between triglyceride glucose-body mass index and cardiovascular outcomes in patients undergoing percutaneous coronary intervention: a retrospective study", Cardiovascular Diabetology, 2023 — 16 words — < 1%
Crossref

19 cardiab.biomedcentral.com — 16 words — < 1%
Internet

20 Pravin Sahadevan, Vineet Kumar Kamal, Akhil Sasidharan, Bhavani Shankara Bagepally, Dolly Kumari, Anita Pal. "Prevalence and risk factors associated with undiagnosed diabetes in India: Insights from NFHS-5 national survey", Journal of Global Health, 2023 — 16 words — < 1%
Crossref

21 Ting Wang, Seidu A. Richard, Junrao Li, He Jiao, Changwei Zhang, Chaohua Wang, Sen Lin, Xiaodong Xie, Chao You. "Cerebral vasospasm resulted in “stent shortening” after pipeline assisted coil embolization for blood blister aneurysms", Medicine, 2021 — 16 words — < 1%
Crossref

22 jmhg.springeropen.com — 16 words — < 1%
Internet

23 Qiang Chen, Shiqiang Xiong, Tao Ye, Yanxiang Gao et al. "Insulin resistance, coronary artery lesion complexity and adverse cardiovascular outcomes in patients with acute coronary syndrome", Cardiovascular Diabetology, 2024 — 15 words — < 1%
Crossref

24 repository.ubn.ru.nl — 15 words — < 1%
Internet

25 Yan Ling, Ding Shuang, Gu Bing, Ma Ping. "Clinical application of simultaneous detection of cystatin C, cathepsin S, and IL-1 in classification of coronary artery disease", The Journal of Biomedical Research, 2017 — 13 words — < 1%
Crossref

26 academic.oup.com — 13 words — < 1%
Internet

27 core-cms.prod.aop.cambridge.org — 13 words — < 1%
Internet

28 hdl.handle.net — 13 words — < 1%
Internet

29 Binyang Yu, Min Li, Zongliang Yu, Tao Zheng, Xue Feng, Anran Gao, Haoling Zhang, Rui Gao. "The Non-High-Density Lipoprotein Cholesterol to High-Density Lipoprotein Cholesterol Ratio (NHHR) as a Predictor of All-Cause and Cardiovascular Mortality in US Adults with Diabetes or Prediabetes: NHANES 1998-2018", Research Square Platform LLC, 2024 — 12 words — < 1%
Crossref Posted Content

30 Duan Wang, Li Ming, Yong Zhu. "Antenatal corticosteroid administration-to-birth interval and — 11 words — < 1%

neonatal outcomes in very preterm infants: A secondary analysis based on a prospective cohort study", PLOS ONE, 2023
Crossref

---

31 Fan Zhu, Wenyuan Gan, Hui Liu, Wenli Chen, Xingruo Zeng. "Risk Factors for Renal Function Progression in Patients with CKD Complicated by COPD", Springer Science and Business Media LLC, 2024 — 11 words — < 1%
Crossref Posted Content

---

32 Ying Song, Weiting Cai, Lin Jiang, Jingjing Xu et al. "Effect of high sensitivity C-Reactive Protein on Uric Acid-related Cardiometabolic Risk in Patients with Coronary Artery Disease— A Large Multicenter Prospective Study", Cold Spring Harbor Laboratory, 2024 — 11 words — < 1%
Crossref Posted Content

---

33 www.yumpu.com — 11 words — < 1%
Internet

---

34 1library.net — 10 words — < 1%
Internet

---

35 Wei Zhang, Min Yang, Mei Zhou, Yuanlang Wang, Xudong Wu, Xiaodong Zhang, Yueyun Ding, Guiying Zhao, Zongjun Yin, Chonglong Wang. "Identification of Signatures of Selection by Whole-Genome Resequencing of a Chinese Native Pig", Frontiers in Genetics, 2020 — 10 words — < 1%
Crossref

---

36 Weipeng Li, Chaonan Shen, Weiya Kong, Xiaohui Zhou, Huimin Fan, Yuzhen Zhang, Zhongmin Liu, Liang Zheng. "Association between the triglyceride glucose-body mass index and future cardiovascular disease risk in a population with Cardiovascular-Kidney-Metabolic syndrome stage 0–3: a nationwide prospective cohort study", Cardiovascular Diabetology, 2024 — 10 words — < 1%

Crossref

37 bmcpublichealth.biomedcentral.com
Internet
10 words — < 1%

38 pubmed.ncbi.nlm.nih.gov
Internet
10 words — < 1%

39 www.bcis.org.uk
Internet
10 words — < 1%

40 Yue-Yang Zhang, Bing-Xue Chen, Qin Wan. "Non-linear association between the plasma atherogenic index and type 2 diabetes mellitus: findings from a multicentre prospective cohort study based on the 4C study", Springer Science and Business Media LLC, 2024
Crossref Posted Content
9 words — < 1%

41 cyberleninka.org
Internet
9 words — < 1%

42 dmsjournal.biomedcentral.com
Internet
9 words — < 1%

43 www.nature.com
Internet
9 words — < 1%

44 Andrew Baum, Tracey A. Revenson, Jerome Singer. "Handbook of Health Psychology - Second Edition", Psychology Press, 2019
Publications
8 words — < 1%

45 Jian Han, Weifeng Dai, Lixia Chen, Zhenhua Huang, Chengzhi Li, Keke Wang. "Elevated triglyceride-glucose index associated with increased risk of diabetes in non-obese young adults: a longitudinal retrospective cohort study
8 words — < 1%

from multiple Asian countries", Frontiers in Endocrinology, 2024
Crossref

46 Sehoon Park, Kyungdo Han, Soojin Lee, Yaerim Kim et al. "Cardiovascular or mortality risk of controlled hypertension and importance of physical activity", Heart, 2021 — 8 words — < 1%
Crossref

47 Wilbert S. Aronow, Jerome L. Fleg, Michael W. Rich. "Tresch and Aronow's Cardiovascular Disease in the Elderly", CRC Press, 2019 — 8 words — < 1%
Publications

48 "Abstracts", Catheterization and Cardiovascular Interventions, 2007 — 7 words — < 1%
Crossref

49 Antonio M. Gotto, Peter P. Toth. "Comprehensive Management of High Risk Cardiovascular Patients", CRC Press, 2019 — 7 words — < 1%
Publications

50 H.-M. Yao, Y.-D. Wan, X.-J. Zhang, D.-L. Shen, J.-Y. Zhang, L. Li, L.-S. Zhao, T.-W. Sun. "Long-term follow-up results in patients undergoing percutaneous coronary intervention (PCI) with drug-eluting stents: results from a single high-volume PCI centre", BMJ Open, 2014 — 7 words — < 1%
Crossref

51 Hezeng Dong, Zhaozheng Liu, Hao Chen, Jin Ba, Rui Shi, Qu Jin, Xiao Shao, Tenghui Tian, Jinzhu Yin, Liping Chang, Yue Deng. "Association between glycemia and multi-vessel lesion in participants undergoing coronary angiography: a cross-sectional study", Frontiers in Cardiovascular Medicine, 2024 — 7 words — < 1%
Crossref

52 Zenglei Zhang, Lin Zhao, Yiting Lu, Xu Meng, Xianliang Zhou. "Association between non-insulin-based insulin resistance indices and cardiovascular events in patients undergoing percutaneous coronary intervention: a retrospective study", Cardiovascular Diabetology, 2023
Crossref
7 words — < 1%

EXCLUDE QUOTES	OFF	EXCLUDE SOURCES	OFF
EXCLUDE BIBLIOGRAPHY	ON	EXCLUDE MATCHES	OFF
