# Supplementary material for: Association between the non-high-density lipoprotein cholesterol to high-density lipoprotein cholesterol ratio (NHHR) and cardiovascular outcomes in patients undergoing percutaneous coronary intervention: a retrospective study
Source: Lipids Health Dis. 2024 Oct 1;23:324. doi: 10.1186/s12944-024-02309-4 (PMC11443751; doi:10.1186/s12944-024-02309-4)
Supplement: Supplementary file 4 — Supplementary Material 4 [file 12944_2024_2309_MOESM4_ESM.doc]

| Supplementary Table 1 Univariate analysis for incidence of MACCEs. | | |
| --- | --- | --- |
| Variable | OR(95%CI) | P-value |
| Age(years) | 1.01 (1~1.02) | 0.045 |
| Sex,n(%) |  |  |
| Female | Reference |  |
| Male | 1.28 (0.96~1.69) | 0.092 |
| Smoking,n(%) |  |  |
| No | Reference |  |
| Yes | 1.03 (0.78~1.34) | 0.851 |
| Clinical presentation,n(%) |  |  |
| STEMI | Reference |  |
| NSTE-ACS | 1.07 (0.72~1.6) | 0.738 |
| SA | 0.86 (0.64~1.15) | 0.308 |
| LVEF, **Mean ± SD** | 1.00 (0.98~1.02) | 0.946 |
| Heart Failure,n(%) |  |  |
| No | Reference |  |
| Yes | 1.47 (1.03~2.1) | 0.032 |
| Atrial Fibrillation,n(%) |  |  |
| No | Reference |  |
| Yes | 2.14 (1.05~4.37) | 0.037 |
| Prior MI,n(%) |  |  |
| No | Reference |  |
| Yes | 1.02 (0.67~1.56) | 0.936 |
| Stroke,n(%) |  |  |
| No | Reference |  |
| Yes | 1.64 (1~2.67) | 0.048 |
| Prior stroke,n(%) |  |  |
| No | Reference |  |
| Yes | 1.21 (0.94~1.56) | 0.148 |
| DM,n(%) |  |  |
| No | Reference |  |
| Yes | 1.09 (0.81~1.48) | 0.567 |
| Creatinine(umol/L) | 1.00 (1.00~1.00) | 0.853 |
| Glycemia(mmol/L) | 1.02(0.92~1.28) | 0.152 |
| TC(mmol/L) | 1.09 (0.97~1.22) | 0.167 |
| TG(mmol/L) | 1.04 (0.96~1.13) | 0.342 |
| HDL-c(mmol/L) | 0.9 (0.59~1.38) | 0.639 |
| LDL-c(mmol/L) | 1.08 (0.94~1.23) | 0.284 |
| Aspirin,n(%) |  |  |
| No | Reference |  |
| Yes | 1.78 (0.42~7.55) | 0.431 |
| Clopidogrel,n(%) |  |  |
| No | Reference |  |
| Yes | 1.92 (0.19~19.94) | 0.584 |
| β-blocker,n(%) |  |  |
| No | Reference |  |
| Yes | 1.05 (0.78~1.41) | 0.742 |
| Statin,n(%) |  |  |
| No | Reference |  |
| Yes | 0.92 (0.55~1.53) | 0.746 |
| Multi Vessel,n(%) |  |  |
| No | Reference |  |
| Yes | 1.58 (1.19~2.08) | 0.001 |
| LM,n(%) |  |  |
| No | Reference |  |
| Yes | 1.68 (0.91~3.1) | 0.1 |
| LAD,n(%) |  |  |
| No | Reference |  |
| Yes | 1.19 (0.84~1.7) | 0.322 |
| LCX,n(%) |  |  |
| No | Reference |  |
| Yes | 1.37 (1.06~1.77) | 0.015 |
| RCA,n(%) |  |  |
| No | Reference |  |
| Yes | 1.24 (0.96~1.6) | 0.102 |
| Occulsion,n(%) |  |  |
| No | Reference |  |
| Yes | 1.29 (0.91~1.83) | 0.158 |
| CTO,n(%) |  |  |
| No | Reference |  |
| Yes | 2.11 (1.46~3.06) | <0.001 |
| Ostio lesion,n(%) | 1.2 (0.81~1.76) | 0.365 |
| No |  |  |
| Yes |  |  |
| Bifurcation lesion,n(%) | 0.98 (0.7~1.37) | 0.916 |
| No |  |  |
| Yes |  |  |
| Restenosis,n(%) | 1.93 (0.78~4.8) | 0.154 |
| No |  |  |
| Yes |  |  |
| Length of stent(mm) | 1.01 (1~1.01) | 0.005 |
| Diameter of stent(mm) | 0.52 (0.38~0.72) | <0.001 |

**Note:** HF, heart failure, AF, atrial fibrillation; LVEF, left ventricular ejection fraction; STEMI, ST-segment myocardial infarction; NSTE-ACS, non-ST elevation acute coronary syndromes; SA, stable angina; MI, myocardial infarction; TC, total cholesterol; TG, triglyceride; HDL-C, high density lipoprotein cholesterol; LDL-c, low density lipoprotein cholesterol; LM, left main coronary artery; LAD, left anterior descending; LCX, left circumfex artery; RCA, right coronary artery; CTO, chronic total occlusions; *P* values in bold are<0.05
