# Supplementary material for: Association between the non-high-density lipoprotein cholesterol to high-density lipoprotein cholesterol ratio (NHHR) and cardiovascular outcomes in patients undergoing percutaneous coronary intervention: a retrospective study
Source: Lipids Health Dis. 2024 Oct 1;23:324. doi: 10.1186/s12944-024-02309-4 (PMC11443751; doi:10.1186/s12944-024-02309-4)
Supplement: Supplementary file 1 — Supplementary Material 1 [file 12944_2024_2309_MOESM1_ESM.doc]

Subject: Response to Manuscript ID b3fdbb1b-55f8-41fb-a02c-f09d54f7b2ad Review

Dear Dr. Abd El-Aty,

Thank you for your prompt review and feedback on our manuscript entitled "Association between the non-high-density lipoprotein cholesterol to high-density lipoprotein cholesterol ratio (NHHR) and cardiovascular outcomes in patients undergoing percutaneous coronary intervention：a retrospective study."

We appreciate the time and effort invested by you and the reviewers. In response to the comments and suggestions, we have taken the following actions:

1. We have run the manuscript through iThenticate, and the similarity index has been significantly reduced to meet the journal's requirements. The proof report is attached as Supplementary Material.

2. A native English speaker has carefully reviewed the manuscript for grammar and syntax errors. We have uploaded a certificate of language editing as Supplementary Material.

3. The reference lists and the manuscript have been meticulously prepared according to the journal format, ensuring that references in the text run seamlessly.

4. Abbreviations have been fully stated before being abbreviated throughout the manuscript.

5. The p letter for statistical analysis has been corrected to uppercase and italic face throughout the manuscript.

6. We have revised the manuscript to use impersonal phrasing throughout, eliminating the use of "we" and "our."

7. The hypothesis and novel aspects of the study are now clearly stated in the introduction.

8. A separate section highlighting the study's strengths and limitations has been included before the conclusion, starting with the strengths followed by limitations.

9. The clinical relevance of the study has been added to the conclusion section, providing insights into how our findings impact patient care.

10. Tables, figures, and references have been cited in order without any omissions.

11. Table legends and figure captions have been provided without missing any.

12. Continuous line numbering has been inserted throughout the text.

13. Spaces and punctuation have been carefully checked and corrected throughout the manuscript.

14. We have prepared a comprehensive cover letter addressing the resubmission of the rejected manuscript, responding point by point to the reviewers' comments, and outlining the amendments and justifications made.

We believe these revisions address the concerns raised, and we are interested in resubmitting the corrected manuscript. We sincerely hope these changes align with the standards of Lipids in Health and Disease.

Thank you for your consideration, and we look forward to your guidance on the next steps in the submission process.

Kind regards,

[Hang Zhao]

[E-mail: zhaohang17777@163.com]
